# Indigenous pregnancy: Agency and strength of Batwa women challenging colonialism and gender inequity

**DOI:** 10.1371/journal.pgph.0005809

**Published:** 2026-01-28

**Authors:** Kaitlin Patterson, Shuaib Lwasa, Lea Berrang-Ford, Seungmi Yang, Jan Sargeant, Charity Kesande, Batwa Communities, Sabastian Twesigomwe, Sherilee L. Harper

**Affiliations:** 1 Department of Population Medicine, University of Guelph, Guelph, Ontario, Canada; 2 Department of Geography, Geo-Informatics and Climatic Sciences, School of Forestry, Environmental and Geographical Sciences, College of Agricultural and Environmental Sciences, Makerere University, Kampala, Uganda; 3 Priestley International Centre for Climate, University of Leeds, Leeds, United Kingdom; 4 Department of Epidemiology, Biostatistics and Occupational Health, Purvis Hall, McGill University, Montreal, Quebec, Canada; 5 Batwa Development Program, Buhoma, Uganda,; 6 School of Public Health, Edmonton Clinic Health Academy, University of Alberta, Edmonton, Alberta, Canada; PLOS: Public Library of Science, UNITED STATES OF AMERICA

## Abstract

Pregnancy and birth can be times of joy, hope, ceremony, and connection for Indigenous women. However, Indigenous maternal health and wellbeing are adversely affected by colonialism and socioeconomic inequities, resulting in otherwise preventable maternal morbidity and mortality. These complex inequities (e.g., marginalization, economic barriers, gendered violence) are highly context specific, and understanding these contexts is essential to inform actions to improve Indigenous maternal health.This study examined the contexts shaping Batwa women’s maternal health in Kanungu District, Uganda, and explored how women engage with, challenge and define their own pregnancy and birth experiences. Using a community-based research approach, we partnered with Batwa women and their communities. We conducted 12 focus group discussions with 44 Batwa women and 16 men across three communities, 49 in-depth repeat interviews with 10 Indigenous Batwa women who had experienced pregnancy, and 17 interviews with 22 maternal healthcare providers. Data were analysed using a constant comparative method and thematic analysis. Four themes related to Indigenous pregnancy were identified: gendered pregnancy expectations; gender roles during pregnancy and birth; gender discrimination and violence during pregnancy and birth; and Indigenous resilience and resistance. Batwa women described pregnancy and childbirth as both joyous and an onerous expectation. Most women’s experiences were characterized by limited partner support, and many included instances of domestic and institutional violence. Women resisted challenges throughout their pregnancies and births finding comfort and strength in Indigenous knowledge and ceremony. These findings demonstrate that Indigenous Batwa women’s pregnancy and birth experiences are profoundly shaped by intersecting gender inequities and colonial structures that continue to undermine their safety, autonomy and access to care. Addressing these inequities requires more than improving service availability; it demands structural reforms that confront gendered violence, eliminate discriminatory practices in health facilities, and meaningfully include Indigenous leadership in maternal health policy and programming.

## 1. Introduction

Pregnancy and birth are often times of joy, hope, ceremony and connection for Indigenous women [1]. Colonialism through oppression, displacement, and violence has disrupted Indigenous maternal health knowledges and practices, including ways to support safe pregnancies and delivery [2]. For example, Indigenous maternal healthcare and midwifery have been criminalized or banned in some countries, while in other places, pregnant Indigenous women may not have access to Indigenous knowledge, medicines, and resources to ensure safe births within their communities due to the imposition of Western medicine and ongoing colonialism [2–4]. At the same time, Indigenous women’s interactions with biomedical health care systems are systematically poorer than that of other groups, whereby they receive lower-quality and/or abusive or inappropriate care [2,5]. Indigenous women have responded to these challenges by relying on family and communities for support, as well as reclaiming Indigenous birthing practices [1]. They also seek high-quality, culturally safe maternal health care that meets their needs, and may reject lower-quality care options, even if this means avoiding biomedical care altogether [2,6–8].

In Africa, when nation states regained independence many of the political and social institutions that transferred were built and rooted in colonial structures [9]. Colonialism in Africa is complex and is characterised by both the impacts of European colonialism and the ongoing marginalization of Indigenous Peoples by African nation states. For many Indigenous Peoples, this, in part, results in a lack of recognition and acknowledgement [9]; for instance, while Batwa are recognized as Indigenous Peoples by neighbouring populations and internationally by the United Nations [10], they have not been recognized as Indigenous in Ugandan law [11]. European colonial rule in Uganda intensified gender inequities by selectively expanding opportunities for men, especially in education and wage labour, while restricting women’s access to resources, mobility and decision making power [12]. These gendered exclusions shaped women’s health by undermining their autonomy, reducing access to biomedical and traditional care, and marginalizing Indigenous knowledge systems. As women navigated these constraints, many engaged in informal economic activities as acts of necessity, resilience and resistance to the narrow roles imposed on them [12]. These inequities did not disappear with independence. Instead, political and health systems continued to operate within colonial frameworks that reinforced social hierarchies, constrained women’s choices and failed to meaningfully center Indigenous women’s reproductive needs [13,14]. Like many Indigenous women across Africa, Batwa women now experience the intersecting effects of gender discrimination, colonial legacies, and ongoing systemic marginalization, conditions that profoundly shape their maternal health experiences today [5,9,15] (we provide additional history and current context of the Batwa in more detail in the methods section).

Examining the impact of colonialism and gender inequity on maternal health of Indigenous Batwa women is particularly relevant in light of their continued high burden of illness [16,17] and in the barriers they continue to face in accessing maternal health care [18–20]. While there is general agreement in the published literature on the drivers of maternal outcomes, there has been little consideration of how these may vary for Indigenous People(s) [5,16,21,22]. Further, global attempts to improve maternal health have largely focused on increasing access to maternal health services [23] without meaningfully addressing how women are treated once they arrive at health centers [24–27].

Indigenous maternal health and wellbeing are intricately linked to discrimination, as well as gender and economic inequities, leading to otherwise preventable illness and death [6,27–30]. These inequities are not only structural but also enacted in everyday interactions with health systems, where discrimination manifests through gender norms that devalue women’s health, economic barriers that restrict timely access to services, racism that positions Indigenous peoples as inferior, and cultural and linguistic differences that delegitimize Indigenous knowledge systems [31,32]. For example, research has documented how Indigenous women are shamed for using traditional medicines, denied care due to inability to pay, subjected to verbal and physical abuse during labour, or mistreated based on stereotypes of being “uneducated,” “weak,” or “non-compliant” [24,26,30]. Such experiences of obstetric violence and systemic exclusion not only erode trust in biomedical institutions but also drive Indigenous women to avoid or resist formal maternal health services, with significant consequences for maternal and newborn health outcomes [5,33]. Given the high prevalence of pregnancy loss among Indigenous Batwa women [17] and the challenges they face in accessing and using maternal healthcare [18–20,34], there is a need to better understand the contexts that shape Batwa maternal health and wellbeing [5,32,35]. Additionally, it is imperative to examine the ways in which Indigenous Batwa women challenge these realities and reclaim and define their own pregnancies and births. This aligns with global calls to document not only the risks, challenges and barriers of Indigenous Peoples, but also the ways they actively navigate, resist and transform oppressive structures [36,37]. Scholars have shown that focusing solely on risks and deficits can perpetuate stereotypes of passivity, whereas highlighting resistance and strength acknowledges women’s leadership in protecting both the health of their pregnancy and themselves [28,38–41].

## 2. Methods

The development, implementation, and interpretation of this research project were guided by six principles for meaningful community-based research: (1) acknowledging and addressing the imbalances of power between the research team and community partners; (2) focusing research on important community issues; (3) fostering empowerment; (4) developing community capacity and building from existing capacity and strengths; (5) working meaningfully with community members as partners; and (6) respecting established protocols for working with Indigenous Peoples [42]. The importance of researching maternal health and Batwa’s experiences was highlighted by the communities during work done by the research team on food insecurity that began in 2009. In 2016, when the research team was developing new grant proposals we met with community members to co-develop objectives, research questions and select methodologies to better understand Batwa maternal health. Recruitment for participants began 15/07/2017 and finished 20/12/2017. This manuscript adheres to the Consolidated Criteria for Reporting Qualitative Research (COREQ) guidelines for reporting qualitative research (S1 COREQ Checklist in S1 File).

### 2.1 Positionality statement

This research centers Batwa women’s voices, with Batwa women and communities as co-authors and partners throughout the project. The research team included one Indigenous Batwa woman, 10 Indigenous Batwa communities, one Ugandan Professor, one Ugandan Healthcare worker, three non-Indigenous Canadian professors and one non-Indigenous Canadian PhD Student. The teams’ diverse perspectives were critically reflected upon to navigate power dynamics, gender inequities, colonialism and ethical responsibilities.

Recognizing that research has historically exploited Indigenous and African communities, the project prioritized partnership and accountability. Batwa partners guided all aspects of research, from design to dissemination, ensuring that methods respected cultural norms and community priorities. A strength-based approach was used to document maternal health inequities while avoiding deficit-based framing that reinforces stereotypes. Indigenous knowledge, lived experience, and scholarship from Black, African, and Indigenous authors informed the interpretation of findings, highlighting both challenges and resilience.

This collaborative, reflexive approach sought to center Batwa voices, challenge colonial structures, and produce knowledge that is accountable, ethical, and actionable. Co-authorship and partnership across diverse identities were essential to ensuring that the research process respected community expertise, promoted shared authority, and contributed to equitable maternal health knowledge.

In this manuscript we use the term colonialism intentionally, rather than “neocolonialism” or “legacies of colonialism”. This reflects the position of Batwa partners and many Indigenous scholars globally, who emphasize that colonialism is not a past event but an ongoing structure that continues to shape Indigenous Peoples’ lives, lands and health [43,44]. To frame these realities as “legacies” risks implying that colonialism has ended, whereas Batwa women continue to experience direct impacts of displacement, non-recognition by the Ugandan state [11] and ongoing restrictions on access to their ancestral lands.

### 2.2 Context: Batwa women of Kanungu district

Indigenous Batwa have lived in forest environments for thousands of years. In Uganda, they were forcefully evicted from their lands when Bwindi Impenetrable Forest and Mgahinga Gorilla National Parks were established in 1991 [45]. The forest was, and continues to play, a key role in the health of Batwa, providing Indigenous medicines, foods, and spiritual connections. Continued barriers to accessing the forest for Batwa are deteriorating their knowledges, deeply felt connections, and ability to maintain harmony between the forest and their communities [15]. However, Batwa are resisting this dispossession by engaging in oral histories, advocating for access to the forest, and ensuring Indigenous knowledge is being passed on to Batwa youth [46].

Indigenous Batwa women experience inequities rooted in marginalization, discrimination, and settler colonialism [47]. Social, economic, and health inequities are well documented between non-Batwa and Batwa communities in Kanungu District [48,46]. Further, gender inequity impacts Batwa women through multiple avenues including domestic violence that has increased substantially with new sedentary livelihoods outside of the forest [15]. Research in the District has documented high prevalence of pregnancy loss [17], low-birth weight infants [18], malaria [49], acute gastrointestinal illness [50], food insecurity [51], and malnutrition [52] among the Batwa; all of these health outcomes are strongly embedded within social, economic, and environmental inequities. However, the focus on marginalization and vulnerability perpetuates stereotypes and can obscure the strength and agency of Batwa women. Herein, we aim not to minimize women’s experiences but to highlight their resilience and agency in spite of these challenges [53].

### 2.3 Data collection

Batwa community members specified their preference for focus groups during early consultations. Women said they liked focus groups because they were fun, interesting to learn from others, and felt safe because there was no pressure to answer questions. A series of three focus group discussions were held in three Batwa communities (n = 9 focus group discussions) (Table 1), reflecting varying experiences of rurality and access to maternal health care services in the District. The focus groups would typically take place at the community Banda (a building or shelter). The research team in encouraged women to participate in other activities, like crafting, food sorting and preparation, during these sessions to increase comfort of participants, as tactile actions can be comforting, reassuring and offer a moment of intentional pauses and reflection [54]. Overall, the focus groups ranged in size from 5 to 15 participants with a total of 60 unique participants. Women above the age of 18, who were pregnant or had previously given birth were invited to attend the women’s focus groups by the community chair person, while we aimed to have 10 or less women, we welcomed anyone who wanted to participate. The topics of these sessions included pregnancy experiences, Indigenous knowledge and practices, and maternal healthcare use. In addition, we held one session in each of these communities with male spouses or partners aged 18 years and over to discuss their perspectives on pregnancy, knowledge, and engagement with maternal healthcare. The focus groups were conducted in English by Patterson and translated in the moment into Rukiga by Kesande and Twesigomwe. The total focus group time was 1320 minutes with an average duration of 110 minutes (range 92–131 minutes) per session. The focus groups were audio recorded with each participant’s informed consent.

**Table 1:** Summary of participants and methods used to examine Indigenous maternal health.

*Location*	*Method*	*Participants*
Community*	*Focus group discussion (n = 60)*	*Batwa community I (n = 15 women; 3 sessions)* *Batwa community I (n = 5 men; 1 session)* *Batwa community II (n = 12 women; 3 sessions)* *Batwa community II (n = 5 men; 1 session)* *Batwa community III (n = 17 women; 3 sessions)* *Batwa community III (n = 6 men; 1 session)*
	*In-depth interview (n = 10)*	*Participant 1 (n = 6 sessions)* *Participant 2 (n = 9 sessions)* *Participant 3 (n = 4 sessions)* *Participant 4 (n = 4 sessions)* *Participant 5 (n = 6 sessions)* *Participant 6 (n = 5 sessions)* *Participant 7 (n = 6 sessions)* *Participant 8 (n = 2 sessions)* *Participant 9 (n = 4 sessions)* *Participant 10 (n = 3 sessions)*
*Health Center or Hospital*	*Interview (n = 22 participants)*	*In-charge of facility (n = 7)* *Medical Officer (n = 2)* *Midwife (n = 12)* *Traditional Birth Attendant (n = 1)*

*Pseudonyms for focus group community and individuals were sometimes used at the request of the participants and in consultation with community chairpeople to protect the confidentiality of experiences shared with the research team.

We also conducted in-depth interviews with individual Batwa women (and one joint interview with their spouse or partner as applicable and appropriate) covering topics such as maternal healthcare and pregnancy experiences, knowledge and use of Indigenous treatments and medicines for maternal wellbeing, and treatment and support of women during pregnancy and post-partum. Women were selected to represent a range of experiences. The research team in collaboration with Batwa leaders contacted five women who were currently pregnant, two women with young children, and three Elder women to share their perspectives and experiences. All ten consented to participate. We met with each woman two to nine times over a 10-week period to develop rapport and provide the space and time to share, in depth, their perspectives on and experiences of maternal health. Interviews were conducted in English and Rukiga, and Twesigomwe and Kesande translated between both languages as needed. The total interview time was 3179 minutes with an average duration of 65 minutes (range 32–104 minutes) per interview.

Finally, seventeen interviews were conducted with 22 maternal health care providers. We interviewed traditional birth attendants and staff from clinics and hospitals that Batwa utilize. Using a semi-structured interview guide with open-ended questions and a conversational tone healthcare providers were asked to share their perspectives on maternal healthcare and health seeking behaviour in Kanungu District. Healthcare workers were invited to participate from facilities that Batwa utilize. Interviews were conducted by Patterson, Kesande, and Twesigomwe in English or Rukiga, depending on the preference of the participant. Each interview was audio recorded with informed consent. The total health care provider interview time was 1108 minutes with an average duration of 65 minutes (range 25–106 minutes) per interview.

### 2.4 Data analysis

The translated English portions of the focus group discussions, in-depth interviews, and maternal healthcare provider recordings were transcribed verbatim by Patterson and checked for accuracy against the audio recordings. To verify the accuracy of translations, an interpreter assessed 5 transcriptions for accuracy and found no major inconsistencies between the recordings and the transcriptions. All finalized transcripts were then prepared for NVivo version 12, for organization and code retrieval during thematic analysis (QSR International Pty LtD, 2018). This analysis involved several iterative steps. First, Patterson engaged in data familiarization and immersion by reading and re-reading transcripts and listening to the recordings. Preliminary codes were then generated inductively by Patterson, capturing both descriptive content (e.g., interactions with healthcare providers) and interpretive dimensions (e.g., resistance, discrimination). These initial codes were discussed collectively and refined into a codebook. Next coded segments were reviewed and organized into broader categories by identifying similarities, overlaps and patterns across women’s individual and group interviews and the men’s and healthcare provider narratives. We used the constant comparative method throughout this stage, iteratively comparing data within and across transcripts to refine codes, explore contrasts, and deepen conceptual coherence. Categories were then iteratively grouped into candidate themes that capture more conceptual patterns in the data. Themes were then refined and redefined through repeated comparison within and across transcripts and through regular peer debriefings (within research team) and community review. Our thematic analysis was guided by Braun and Clarke’s [55,56] six-phase approach and further informed by Vaismoradi et al.’s [57] description of moving codes to themes in qualitative content analysis. In addition, our community-based approach and Indigenous research principles [17] shaped interpretation by centering Batwa women’s voices, privileging Indigenous knowledge and incorporating community feedback into theme development.

Peer debriefing was conducted to improve the rigour and validity of data collection and interpretation [58]. After each interview or focus group discussion, Patterson, Kesande and Twesigomwe met for recorded debrief sessions (652 recorded minutes). These recordings provided context, and an Indigenous lens through which to understand the stories, experiences, and perspectives being shared with the research team. Finally, the presentation and interpretation of these narratives, experiences, and perspectives was done in partnership with, and the permission of all the Batwa women involved. In some circumstances, pseudonyms were used and identifying personal information and details were altered to keep their identity confidential at the participant’s request.

### 2.5 Ethics and inclusivity in global research

Along with Batwa community members, we co-developed a consent process, that included an initial consent to participate acknowledging that consent is a relationship and an ongoing decision [59,60]. Ethics approval was obtained from Ugandan and Canadian Institutions: University of Guelph Research Ethics Board, Makerere University School of Social Sciences Research Ethics Committee, and the Uganda National Council for Science and Technology. Preliminary results were shared in person with Batwa communities in Uganda in 2017. Summarized results and the manuscript were shared with Batwa community members via WhatsApp in 2020 and 2021, and in person results were shared with each community in 2022. Additional information regarding the ethical, cultural, and scientific considerations specific to inclusivity in global research is included in the Supporting Information (S1 Checklist)

While there is variation in approaches and theories on compensation in research, it was important that we acknowledged and valued the time Indigenous women and their respective communities were spending with us and recognized the contributions they were making to the research [61]. As a result, we negotiated with each of the in-depth interview participants on fair compensation. We also worked with communities to identify an appropriate thank you for participants in the focus group discussions (most communities opted for meat, and household goods) [61]. Chair people were paid for their time reaching out to participants, time with the research team, and for organizing community spaces and gatherings. Finally, maternal health providers (where they were interviewed during work time were not paid) were given a token of appreciation.

## 3. Results

The research findings are organized into themes identified from the data that characterize Indigenous Batwa pregnancy and birth: gendered pregnancy expectations; gender roles during pregnancy and birth; gender discrimination and violence during pregnancy and birth; and Indigenous resilience and resistance. These results center around Batwa women’s voices and experiences, highlighting the joys and challenges that shape their pregnancies and births.

### 3.1 Gendered pregnancy expectations

All participants stated that “*producing”* or birthing children is required of women, but all participants also highlighted the joys of having a child, “*having a baby, having a child it’s like a blessing, because if you have a baby in your life you are happy” (focus group).* Women said they need to have children to have “*value*” and to be accepted by their husband and community. As a Batwa participant explained a female only becomes a “*woman*” and only gains an “*identity*” once she has produced a child, “*because when you do not have a child, you don’t have an identity, people hardly recognize you” (Imelda).* Further, women stated they must produce multiple children to maintain their social value. As one Batwa woman explained, “*having like one child or two, you aren’t that respected in the community. They are like, now which type of lady is this with just one child?” (Beatrice).* Women explained that not having children or losing fertility exposed women to domestic violence and/or divorce or loss of support from their spouse. Constance explained, “*when I am not pregnant, we [Constance and spouse] can even start quarrelling, he is always complaining, why have you delayed, why are you delaying getting pregnant, I want another child.”* Men generally reported wanting many children, while women reported that men rarely considered the impact of pregnancy and birth on women’s health and/or the workload of raising children*.*

For Indigenous Batwa women, pregnancy and birth was also associated with ensuring the survival of the Batwa People. Many participants expressed the fear that Batwa would disappear, when they were discussing their experiences of infant and child loss. Some of the Batwa men’s focus group discussion participants explained “*the death rate of the Batwa is very high compared to other tribes [ethnic groups]. So that’s why we opt to have many children. I had ten [children], now only four are remaining [haven’t passed away]. So, if I had only produced four, I would be having nothing.”* Both men and women reported the importance of “*enlarging the tribe*.” Some Batwa men explained “*because our tribe, our clan is the minority, we want to expand*” and “*you have to leave somebody [a legacy] behind*.”

This imperative to have children was a key reason that women reported avoiding family planning and contraception. Contraception was conceptualized as “*family planning*” in Kanungu District and was viewed by participants as a way for married women to space their pregnancies and manage family size. Generally, men reported that they did not support their wives using family planning. Some women who did use family planning said they did it in secret, which at times led to family conflict and domestic violence. A healthcare worker explained “*they [women] are getting family planning services, without their husband’s knowledge, and they keep on hiding their husbands, cause some husbands, if they came to know, we find they’re blaming and beating them.”* Other women who did have permission from their spouse to use family planning reported other challenges, including access to transportation, adequate free time, lack of contraception at health centers, and religious barriers.

### 3.2 Gender roles during pregnancy and birth

Since their eviction from the forest, Batwa women described how they have become the primary food providers for their household. Batwa women said they are expected to acquire and prepare all the food for the household, in addition to collecting firewood and water, child rearing, and maintaining gardens and crops. Most participants spoke of food insecurity and women said they are often unable to acquire sufficient food for their households. Women explained how their responsibilities do not change during pregnancy or postpartum, “*you can’t finish all those months [of pregnancy] just seated and eating*,” unless you were “*sick*,” you “*had to keep working*.” Most Batwa women referred to agricultural labour, or “*digging*,” under the employment of non-Batwa as their primary form of work. Women described maintaining work and household responsibilities while pregnant as extremely difficult and stressful. Caloric needs increase during pregnancy and postpartum if breastfeeding, however, Batwa women said that because they felt “*very weak*” during pregnancy they often acquired less food than normal for their children, spouses, and themselves. Pregnancy loss (miscarriage and stillbirth) was also brought up by women as a concern linked to overworking, lifting heavy things, and working in the sun and the heat. During antenatal care, Batwa women said they are told to reduce the amount of work they are doing and to limit “*heavy work*” (e.g., agricultural digging, and collection of firewood and water). One woman explained, “*I listen, but because I don’t have food, I have to keep doing that heavy work.”* Most women said they worked until their contractions started, and even then, some women still went to work until they had “*overwhelming contractions*,” because they feared their family would go without food when labour may have been a false alarm. Postpartum, women said they are usually permitted a few days for recovery: if they had a vaginal birth usually about a week, if they had a c-section, about 3 weeks. One participant explained that women were terrified of having c-sections because “*when you have an operation you become lame and you will no longer be able to do heavy activities, you can no longer do activities you have been doing and you become so weak compared to how you used to be*”.

Women reported varying levels of support from children, family, friends, and spouses during pregnancy and birth. As children become older, they reduce the burden of labour placed on women in the household, a Batwa woman shared that “*children can be supportive because when you have a baby you can send older children to fetch water, fetch firewood” (Constance).* Another participant explained, “*If you are sick, they can take care of you, you can go with them in the garden, you can have a big garden” (focus group).* Through children, women said they can increase household food production, have companionship, and gain support systems for when they sick or elderly, particularly if they cannot rely on their spouse for support.

Batwa women also described the support that they get or expect from their spouse during pregnancy, which can comprise financial, emotional, and physical contributions. Some women said their relationships improved during pregnancy because the spouse would help with household chores, contribute to finding food, and do the “*heavy lifting*,” as well as other roles usually assigned to Batwa women. Men also mentioned taking on additional work while their wives were pregnant: one of the men’s focus groups explained that “*when the wife is pregnant, the man takes some of the responsibilities, like digging, because the wife has less energy, she can’t do all those works, so we usually take some of the responsibilities that she has been doing*”. Additionally, there is a social expectation that men provide money and the necessary resources for women to get to a health facility for delivery (transport, delivery supplies, food while the mother is at the hospital). A male participant explained “*now that you have impregnated someone, you should change your lifestyle, now you start the life of a man. You should save and have some money in your pocket in case of anything [birth or pregnancy complication] then you can maybe run and get a boda boda [motorcycle taxi] to the hospital.”* Despite these aspirations, however, many Batwa women explained that spousal or partner savings and financial support for pregnancy and/or delivery were rare.

When women go to a health facility for delivery, they said their sister or mother-in law accompanied them. Men attending deliveries at a health facility is extremely rare, instead they said they would wait at home for their wife to arrive with the new baby. For women delivering at home some spouses play a key role in delivery; one woman whose husband had helped her in previous home deliveries said that “*sometimes men who are brave can help the wife to deliver.”* Other Batwa women described husbands helping guide pushing, providing emotional, and physical support, “*catching the baby,”* cutting the umbilical cord, and disposing of the placenta. But, in a focus group discussion, most Batwa women said their husbands did not help with their home deliveries, saying *“there are men who are cowards, they fear, maybe the father can be scared of maybe the wife is dying [during delivery], maybe he doesn’t know how to take care of all that processes, cutting the cord, everything.”*

### 3.3 Gender discrimination and violence during pregnancy and birth

Intimate partner violence (including physical and sexual violence) was frequently discussed as a normalized aspect of marriage and was said to occur for a number of reasons during pregnancy (e.g., alcohol use among spouses, pregnant women wanting unaffordable foods, or being unable to manage household chores). Male focus group participants explained “*we usually quarrel and fight with our wives when they are pregnant*.” Most participants viewed violence during pregnancy as a risk for pregnancy loss. Men in focus groups said they often tried to avoid or reduce violence while their spouse was pregnant because they were worried it may impact the pregnancy. However, some women said that “*husbands don’t think that abusing us will affect our pregnancy*.” Alcohol use seemed to intensify intimate partner violence, with male participants explaining that alcohol impaired their judgement.

Many women also reported experiencing violence and mistreatment during their interactions with health providers at health care facilities. Recurring examples from participants about previous pregnancies included painful vaginal exams, shaming and refusal of service for using herbs and Indigenous medicine, shaming and refusal of service for not shaving pubic hair, and verbal and physical abuse during delivery (e.g., slapping labouring women for making noise, or not pushing). Healthcare providers noted that this “*abusive*” behaviour seemed to be more frequent in maternity wards than in other departments of the health centres and hospitals; as one healthcare provider noted, “*I think maternity is somehow worse than other wards.”* Healthcare providers suggested this treatment of pregnant and delivering mothers by healthcare workers was due to: “*insufficient or poor training,*” small numbers of staff, “*overworking,*” and frustration when patients did not follow medical advice. As one woman explained, “*Because they [healthcare workers] think we are not educated, and when they see us and they are like [you are] not educated, that’s why they are under looking us” (focus group)*. A woman’s status was also suggested as a reason this mistreatment occurs during labour; as one healthcare provider explained, *“[The] big thing is that women here are not empowered to like do anything, and I don’t think they know they can be, that they have like a voice in saying what they want*”*.*

Batwa women often compared their experiences to non-Batwa, describing their insufficient or poor maternal healthcare from health facilities as discrimination. Many Batwa women gave examples of being denied care or being ignored unless they persisted and advocated for care. Hope described her experience while trying to get healthcare for a prolapsed uterus:

*I had a miscarriage in June, and afterwards I continued experiencing heavy pain, so I went to the health centre. So, I explained my experience and afterwards that trainee nurse, “okay, for me I don’t know what’s wrong with you, maybe I go and call someone to examine you.” I was sent to that emergency room, and I waited for some-time, but I never got anyone to come and attend me. So, I got angry with them and just came back home. I went back to the OPD, I told the nurse who had sent me to that emergency room, and she just gave me two pills to swallow and told me to go home. But, I came back, and it’s when I met a good doctor and they examined me and they told me that the uterus was in the wrong position like it was coming out [prolapse] like near the thigh, so they injected me and they pulled out and first cleaned the uterus then and fixed it in the right position. (Agnes)*

Most healthcare workers did not view their interactions with Batwa as discriminatory*.* However, Batwa women reported feeling judged and looked down upon when they sought maternal healthcare. A focus group member described her interactions with health providers saying “*there are some nurses who are there and when they see you, it is as if they want to vomit. They are not smiling at us, they just look like they want to vomit on you, because you are a Batwa” (focus group)*. Some women stated that these experiences or hearing about them deterred them from seeking care and delivering future pregnancies at a health facility. Prossy describes how even though she had a previous c-section, she preferred to deliver at home rather than seek emergency help from a nearby health center after going into labour early:

*I tried to deliver at home, then called the TBA [traditional birth attendant] and she failed [unable to deliver child], I didn’t want to go to the health centre, the nurse is always rude, and abusing us, and angry at people, she speaks rudely to the Batwa, she used to beat the Batwa. – Prossy*

### 3.4 Indigenous resilience and resistance

While negative experiences of poor care, discrimination, and violence were reportedly common, Batwa women demonstrated strength, resilience, and leadership through their choices. Batwa women said they continue to draw on Indigenous medicines and foods for strength and health. Often Batwa said they trusted Indigenous medicines and believed they were more effective than those available through health centers. Indigenous medicines were also an alternative to potentially negative interactions while attempting to seek maternal healthcare at health facilities. A Batwa woman explained, *“We take herbs because we do not trust them [staff at health centers], and they can’t do anything*.” Even women who did seek care at health facilities also practiced Indigenous ceremonies and used Indigenous medicines, sometimes in secret: a Batwa woman described their friend’s experiences at the mother’s waiting hostel, “*she hid behind the kitchen. And she did it [took herbs] when nobody was seeing her.”* A Batwa woman explained that Batwa women staying at health facilities would leave and go to a nearby community to *“do some exercises and take some of the traditional plants.”* Other examples of Indigenous practices described by Batwa women included eating strengthening foods (e.g., honey, wild yams), warding off curses that were placed on them during pregnancy, and protecting infants from harm through a range of physical and spiritual ceremonies. However, in focus groups women explained that their access to medicinal foods and herbs has been drastically reduced since their eviction from the forest. Due to restricted access, the Batwa lamented that some of their knowledge was being lost and that the means of preparation and proper dosing of herbs was being forgotten, potentially increasing the risk in their use.

The birthing process demonstrated the strength and resilience required of Batwa women. When describing how they manage pain during labour most Batwa women said they did not scream, yell, or even make a noise. The following quotes explain why women use silence to demonstrate their strength and resolve:

*For you [western researcher], you scream, because you have support, if anything goes wrong you can be taken to the hospital you can be taken to theatre [for surgery], but for us, because we don’t have money and we don’t have support we have to be brave to push the baby because we don’t have any option. That’s why you scream (Imelda).*

Participants also explained how they identified with being able to *“produce [give birth]”* without help, for them it was an illustration of a lack of support in their day-to-day life and responsibilities. Women found power and strength delivering alone and on their terms.

*I know I am firm and that I can produce alone. I don’t want to people coming because I know I can do it [deliver] alone (Margaret)*

## 4. Discussion

From pre-conception to postpartum, Batwa women carry the primary responsibilities of birthing, raising, and providing for their families, and they described experiencing domestic and institutional violence across these stages. These experiences intersect with colonialism and gender inequity, creating unsafe environments for Indigenous women and complex barriers to accessing maternal health care. Yet, Batwa women also demonstrated agency, control, and multiple forms of resistance as they navigated pregnancy, birth and family planning.

For the Batwa, social determinants of maternal health are shaped by colonial structures. Their current socio-economic position is a direct result of their eviction from the forest [45,62]. The lack of resources in Batwa households kept women from being able to pay for maternal health services or transport and limited the amount of time they were able to seek care when providing for their families. Likewise, previous research has found that substantial disparities relative to other social determinants of health (e.g., education, housing, employment) in relation to non-Batwa women in the District impact Batwa women’s pregnancy outcomes and healthcare use [18–20].

Eviction from the forest has also had a direct impact on the ability of Batwa women to care for their pregnancies using Indigenous methods. Women’s narratives highlighted loss of access to medicinal foods herbs and plants traditionally used for strength, healing and safe labour. For Indigenous women access to Indigenous remedies and the ability to practice Indigenous medicine is critical to maternal wellbeing [2,31,63,64]. However, many healthcare institutions have demonized the use of Indigenous medicines in pregnancy by framing them as harmful and drawing a distinct line between biomedical and Indigenous practices [31,65]. Continued loss of access to the forest may exacerbate knowledge loss and potentially result in unsafe use of Indigenous medicines, as has been observed elsewhere [66].

Institutional maternal health service encounters underscored the intersection of colonialism, race, and gender inequality for Batwa women. Globally, Indigenous women who give birth at healthcare facilities report experiencing abuse, cultural disconnect, stress, isolation, and “feel dehumanized by the medical encounter” [67, p. 1859]. Likewise, Batwa women reported experiences of verbal and physical abuse and/or poor-quality care at health facilities, during their pregnancies. These encounters were often rationalized by healthcare, as necessary for safe delivery reflecting deeply engrained practices that providers do not realize are discriminatory or harmful [31,68]. Indeed, health providers in our study rationalized the use of physical violence against Batwa women to ensure the safe delivery of their babies, all the while affirming that their actions with the Batwa were not discriminatory. Existing research suggests that when mistreatment is normalized, women’s expectations of care are reduced to medical outcomes rather than quality of care, which reduces their ability to complain or advocate for change [33,68].

Documenting experiences of violence was not a primary objective of this study. However, violence emerged repeatedly in women’s narratives, in community discussions with Batwa women and men, and by maternal healthcare providers. Studies have found that intimate partner violence can cause unintended pregnancies, pregnancy loss, and negative maternal health outcomes including death, and women who experience this violence are less likely to access maternal health care services [69,70]. Colonialism has contributed to increased violence against Indigenous women through altered gender roles, normalized violence in colonial encounters, and the justification of violence as a way to maintain order [5]. In this study, many of the examples women provided when discussing intimate partner violence were linked to the expectation that they produce children and provide and prepare food, and, in other studies, these expectations are closely linked to the Batwa’s eviction from the forest [15]. Jackson [15] has described how the loss of complementary relationships in the forest – where Batwa men and women equally engaged in supporting the household – changed marital relationships, and how the higher mortality rate of children outside the forest has coincided with increased fertility rates among Batwa. Women described that while men often faced consequences for not meeting the expectations of their roles, women who fell short of household and reproductive expectations faced social ostracism, hunger, and increased risk of both institutional and intimate partner violence. These dynamics echo broader evidence linking normalized domestic violence to obstetric violence and mistreatment within health systems [68,71]. These patterns underscore the need for structural interventions that address gender norms, economic marginalization, and colonial inequities that limit Batwa women’s autonomy and wellbeing.

The resilience of Indigenous women is underrepresented in research [37]. Through their discourse and actions, Batwa women demonstrated multiple forms of resistance and agency pertaining to their maternal health experiences. Despite experiences grounded in historical and ongoing oppression, Batwa women navigate these challenges through diverse forms of resistance and adaptation. For example, after leaving the forest women acquired new skills in agriculture and animal rearing, expanded support networks to protect themselves and their children. However, these acts of resilience occurred within significant constraints shaped by gender inequity and colonial histories.

Batwa women described covertly using family planning to protect their health and assert decision-making power, similar to patterns observed elsewhere in sub-Saharan Africa [72]. Women also emphasized self-reliance, including giving birth alone, both as a response to unreliable spousal support and as an expression of strength and independence [73]. Batwa women also emphasized the importance of trusted providers. The story of Agnes illustrates how resilience and trust shaped care-seeking. Finding a culturally safe and trusted healthcare provider is rare for the Batwa. Due to tight community networks among women, others now actively seek him out “*the good doctor*” when they need. Other studies have highlighted that building trust with both individual health providers and institutions is required to improve maternal healthcare for Indigenous women [74]. As demonstrated by Prossy’s experience, abusive interactions in facilities resulted in women refusing to seek even emergency care. Such resistance to harmful care represents an assertion of dignity and highlights how negative encounters damage trust in institutions and maternal health care services more broadly [67].

Emphasizing “*strength*” and “*resilience*” must be done with care. Black and African scholars caution that such narratives can justify structural neglect or normalize violence [75,76], (including in maternity care where women’s pain has been minimized by health care workers [77,78]. These forms of resourcefulness, whether relying on kin networks, reclaiming traditional practices, or refusing discriminatory care, should be recognized as acts of survival within contexts of dispossession, racism and gender-based violence. Resilience frameworks must therefore be balanced with attention to systematic justice to avoid placing the burden of survival on Indigenous women themselves [28,41].

Together, these findings point to critical intervention points to support Batwa women’s agency in maternal health. Increasing facility-based care alone will be insufficient; addressing colonial and gender inequities is essential to reducing gaps in maternal health outcomes [5]. Research on improving care for Indigenous Peoples through partnerships with healthcare institutions has shown that when power imbalances were addressed, trust was developed and fostered [79,80]. Key factors for success in Indigenous maternal health include: (1) incorporation of Indigenous knowledge and practices alongside medical maternal care, (2) the presence of Indigenous healthcare providers, (3) an acknowledgement of colonial trauma, (4) the building of trusting and long-term relationships between Indigenous women and their health provider, (5) institutional accountability, and (6) community level, wholistic interventions such as family counselling and education [5,23,31,56,63,64,80–82]. Without addressing structural inequities that constrain the agency of Indigenous women, preventable maternal mortality and morbidity is likely to continue [5].

This study has several limitations. First, although data were collected in 2017, the structural inequities described remain widely reported; however some contextual changes may have occurred since that time. Second, translation from English and Rukiga may have resulted in minor shifts in meaning despite validation processes. Third, while this study incorporated Batwa men and healthcare providers, our analysis centered Batwa women’s perspectives, and therefore may not fully capture their roles and perspectives. Finally, although community engagement and review strengthened interpretation, power dynamics inherent in research with Indigenous Peoples may have influenced disclosure or the results shared here .

## 5. Conclusion

The narratives shared and presented here underscore how gender inequity, colonialism and resistance shape the experiences of pregnancy for Batwa women. Colonialism and gender inequity are impacting maternal health outcomes through structural gendered expectations, domestic violence, and institutional violence. These findings suggest that measures to support Indigenous women’s agency, dignity, and sovereignty would improve maternal health and the quality of maternal care that they receive. Incorporating Indigenous practices and knowledge into healthcare is essential to disrupting colonial structures that harm Indigenous pregnancies.

## Supporting information

S1 Checklist Inclusivity in global research. (DOCX)

S1 File COREQ Checklist. (PDF)
