# Supplementary material for: Indigenous pregnancy: Agency and strength of Batwa women challenging colonialism and gender inequity
Source: PLOS Glob Public Health. 2026 Jan 28;6(1):e0005809. doi: 10.1371/journal.pgph.0005809 (PMC12851452; doi:10.1371/journal.pgph.0005809)
Supplement: S1 Checklist — (DOCX) [file pgph.0005809.s001.docx]

Inclusivity in global research

PLOS’ policy on inclusivity in global research aims to improve transparency in the reporting of research performed outside of researchers’ own country or community and ensures that PLOS publications reporting global research adhere to high standards for research ethics and authorship. Authors of relevant research articles may be asked to complete the questionnaire below, which outlines ethical, cultural, and scientific considerations specific to inclusivity in global research. This questionnaire may be requested when researchers have travelled to a different country to conduct research, if research uses samples collected in another country, research with Indigenous populations or their lands, or if research is on cultural artefacts. Researchers travelling to another country solely to use laboratory equipment will not normally be required to complete the questionnaire. However, the questionnaire can be requested at the journal’s discretion for any submission – if you have been requested to complete this questionnaire by the PLOS journal you submitted to, please do so.

Please complete the questionnaire below and include this as a Supporting Information file with your manuscript. Note that if your paper is accepted for publication, this checklist will be published with your article in the supporting information files. Please ensure that you reference the checklist in the main body of your manuscript. We suggest adding a subsection ‘Inclusivity in global research’ to your Methods section and adding the following sentence: “Additional information regarding the ethical, cultural, and scientific considerations specific to inclusivity in global research is included in the Supporting Information (SX Checklist)”

The questions have been designed to be applicable to a wide range of study types, and there are subsections for both human subjects research and non-human subjects research. If any of the questions are not relevant to your research please mark them as “N/A” as appropriate.

**Ethical considerations, permits and authorship**

*This section is applicable to all research types.*

Provide details as to who granted permissions and/or consent for the study to take place in the Methods section of your manuscript. This should include the names of **all** ethics boards, governmental organizations, community leaders or other bodies that provided approval for the study. If individuals provided approval refer to these people by their role or title but do not list their name(s).

Reported on page number: 15 in the Ethics and inclusivity in global research sub-section.

Ethical approval for this study was granted by three ethics boards: (1) Makerere Univerity College of Humanities and Social Sciences Research Ethics Committee (2) the Uganda National Council for Science and Technology (UNCST) and (3) University of Guelph Research Ethics Board. As part of community protocols, permission was additionally obtained from Batwa community chair people prior to engaging participants; these individuals are referred to in the manuscript by their leadership roles rather than by name.

If there were any deviations from the study protocol after approval was obtained please provide details of these changes in the Methods section of your manuscript.
Did this study involve local collaborators that are residents of the country where the research was conducted or members of the community studied? If you do not have any authors from said communities, please provide an explanation for this below.

Yes, this study involved 10 community partnerships, Batwa research team members, a Ugandan Professor, Ugandan Healthcare providers, Ugandan research assistants. These collaborators contributed to study coordination, data collection, translation, cultural interpretation, and community engagement. All individuals who met PLOS authorship criteria—including substantial contributions to study design, data collection, interpretation, and manuscript drafting—are included as authors. Individuals who contributed only to logistical support or translation but did not meet authorship criteria are recognized in the acknowledgements.

Reported on page number: N/A

Everyone listed as an author should meet PLOS’ criteria for authorship and all individuals who meet these criteria should be included in the author byline, rather than the acknowledgements. For further information please see the journal’s Authorship Policy.

**Human subjects research (e.g. health research, medical research, cross-cultural psychology)**

Did you obtain written informed consent from a representative of the local community or region before the research took place? How did you establish who speaks for the community? Details of written informed consent obtained from study participants should be reported separately in the Methods section of your manuscript.

Relationships between the research team and Batwa communities have been ongoing since 2010, and maternal health was identified as an important issue by community members during previous collaborative

projects. In 2016, the research team met with Batwa communities and local health providers to co-develop project goals, research questions, and appropriate methods for this maternal health study. Then, in early 2017, several planning meetings were hosted for communities, chairpersons (i.e., elected community leaders), and local leaders to further discuss the project, co-develop approaches to recruit participants, and arrange a data collection calendar. We developed our approach to informed consent in collaboration with communities, which was conceptualized as an ongoing relationship, rather than a one-time request (Baydala et al., 2013).

How did members of the local community provide input on the aims of the research investigation, its methodology, and its anticipated outcome(s)?

Batwa communities were involved in shaping the study throughout its development. During the planning phase, Batwa communities and research team members, and local health workers provided input on the appropriateness of the research aims, the questions guiding the investigation, and culturally acceptable approaches to interviewing and group discussions. Community members also informed the data collection methods, advised on privacy considerations, and contributed to interpretation of findings through follow-up discussions and member-checking sessions.

When engaging with the local community, how did you ensure that the informed consent documents and other materials could be understood by local stakeholders?

All consent forms, information sheets, and interview materials were translated into Rukiga, the primary language spoken in the study communities. Translated documents were developed by local collaborators to ensure accuracy, cultural appropriateness, and readability. During the consent process, researchers read consent documents aloud to participants, provided opportunities for questions, and verified comprehension before written or thumbprint consent was obtained. This process was developed in collaboration with communities. Initially we were only going to record oral consent, however it provided little accountability for the research team. So we created two copies of all documents that each participant could keep their copy and the research team would keep the other.

Will the findings of the research be made available in an understandable format to stakeholders in the community where the study was conducted (e.g. via a presentation, summary report, copies of publications, etc.)? Please provide details of how this will be achieved.

Yes. Findings have been shared with Batwa communities and other partners in collaboratively decided formats. Summary results were shared verbally during community meetings (with mobile projector and via WhatsApp audio messages for those unable to attend in person. As part of ongoing reciprocity commitments, we will provide a short non-technical summary report written in clear English and translated into Rukiga. Copies of the final publication will be provided to local partners, and we will offer to present findings to the District Health Office and community groups to facilitate local application of the results.

**Non-human subjects research using specimens/ animals collected as part of the study, or those housed in archival collections. Examples include archaeology, paleontology, botany and zoology.**

Did the permission you obtained from a local authority to perform the study include an agreement on access to outputs and benefit sharing? This may include procedures to enable fair distribution of the benefits and resources arising from the research performed. Please include any details of Prior Informed Consent and Benefit Sharing Agreements obtained. These may be required by field-specific regulations, for example the Convention on Biological Diversity (CBD) and the associated Nagoya Protocol.

If the material used in your study was imported, please A) provide the year it was imported and B) indicate whether permits were obtained to import/export the materials used, C) provide details of any permits obtained. If this information is not available, please indicate this.

If you used archival specimens, please state how the material used in your study was acquired by the institute it is held in and provide details of any permits obtained for the original excavations/ sample collection. If this information is not available, please indicate this.

How was the potential cultural significance of the materials collected in your study to local communities considered in your research design? Were Indigenous peoples and/or local researchers and institutions involved with archaeological excavations / collection of specimens? If so, please provide a description of their involvement.

If your manuscript includes photographs of human remains please indicate whether authors obtained permission from descendants or affiliated cultural communities to do so.
